# Efficient selective breeding of live oil-rich *Euglena gracilis* with fluorescence-activated cell sorting

**DOI:** 10.1038/srep26327

**Published:** 2016-05-23

**Authors:** Koji Yamada, Hideyuki Suzuki, Takuto Takeuchi, Yusuke Kazama, Sharbanee Mitra, Tomoko Abe, Keisuke Goda, Kengo Suzuki, Osamu Iwata

**Affiliations:** 1 Euglena Co., Ltd., Tokyo 108-0014, Japan; 2 Nishina Center for Accelerator-Based Science, RIKEN, Saitama 351-0198, Japan; 3 Department of Chemistry, University of Tokyo, Tokyo 113-0033, Japan; 4 Department of Electrical Engineering, University of California, Los Angeles, California 90095, USA; 5 Japan Science and Technology Agency, Tokyo 102-0075, Japan

## Abstract

*Euglena gracilis*, a microalgal species of unicellular flagellate protists, has attracted much attention in both the industrial and academic sectors due to recent advances in the mass cultivation of *E. gracilis* that have enabled the cost-effective production of nutritional food and cosmetic commodities. In addition, it is known to produce paramylon (β-1,3-glucan in a crystalline form) as reserve polysaccharide and convert it to wax ester in hypoxic and anaerobic conditions–a promising feedstock for biodiesel and aviation biofuel. However, there remain a number of technical challenges to be solved before it can be deployed in the competitive fuel market. Here we present a method for efficient selective breeding of live oil-rich *E. gracilis* with fluorescence-activated cell sorting (FACS). Specifically, the selective breeding method is a repetitive procedure for one-week heterotrophic cultivation, staining intracellular lipids with BODIPY^505/515^, and FACS-based isolation of top 0.5% lipid-rich *E. gracilis* cells with high viability, after inducing mutation with Fe-ion irradiation to the wild type (WT). Consequently, we acquire a live, stable, lipid-rich *E. gracilis* mutant strain, named B_1_ZFeL, with 40% more lipid content on average than the WT. Our method paves the way for rapid, cost-effective, energy-efficient production of biofuel.

Microalgae-based bioproducts have gained much attention in both the industrial and academic sectors. Microalgae photosynthesize with a higher efficiency than higher plants and can be cultivated in agriculturally unused soils. These features of microalgae have helped us advance the cultivation of microalgae such as *Arthrospira* and *Chlorella* which are nutrient-rich as a source of food for us and other animals^1^. The biomass from mass-cultured microalgae is also exploited as a source of specific chemicals, such as β-carotene, astaxanthin, and polyunsaturated fatty acids, to be used for nutritional supplements, pharmaceutical drugs, and cosmetic products^1^,^2^. Furthermore, many microalgal species are known to produce and store oil in response to nutritional and environmental conditions such as nitrogen deficiency^3^,^4^,^5^. Although there remain a number of technical difficulties to be solved, the economically sustainable production of biofuel from mass-cultured microalgae is highly anticipated as a next-generation energy source^6^,^7^.

*Euglena gracilis*, a species of single-celled flagellate protists found in freshwater, is one of the aforementioned industrially exploited microalgae. *E. gracilis* is neutrient-rich and known to accumulate paramylon (β-1,3-glucan in a crystalline form) as reserve polysaccharide in response to nitrogen deficiency or heterotrophic carbon sources^8^,^9^. Recent reports suggest the functionality of paramylon on hepatoprotection^10^, atopy treatment^11^, and colon cancer suppression^12^. Based on these features, mass-cultured *E. gracilis* has been commercially supplied as an ingredient of functional food and a source of paramylon extraction. *E. gracilis* is also known to consume the intracellular paramylon in a hypoxic condition to obtain energy without oxygen^13^. This process is accompanied by the production of wax ester–an ester mainly composed of C14:0 saturated fatty acid, myristic acid, and myristyl alcohol^14^,^15^. The composition of a relatively short chain of fatty acids and alcohols is especially suited for its conversion to biodiesel and aviation biofuel.

To improve *E. gracilis*’ productivity of the wax ester and further promote the utility of its biomass to biofuel, effective breeding methods need to be developed before it can be deployed in the competitive fuel market. Although a number of *E. gracilis* mutants have previously been developed, most of them have been derived from inefficient mutations in its chloroplast genomes^16^. Furthermore, its killing curve by UV irradiation indicates that *E. gracilis* is polyploid, making it difficult to alter its nuclear genome^17^,^18^. In contrast, heavy-ion irradiation has been identified as an effective approach to producing *E. gracilis* mutants different from those generated by exposing them to classical mutagenesis such as chemical mutagens and UV^19^. Previously, heavy-ion irradiation has been employed for higher plants^20^ and tested on the mutant production of various microalgal species^21^,^22^ and is expected to be of use for the development of *E. gracilis* mutants as well.

Fluorescence-activated cell sorting (FACS) is a powerful tool for counting and characterizing a large heterogeneous population of cells, including microalgal mutants, with a high throughput of more than 10,000 cells/sec by detecting fluorescent light from each laser-excited cell during flow^23^. While FACS is commonly employed in basic medical research and clinical practice, it has also been used to separate a single microalgal species from a mixed culture^24^ and to produce an axenic microalgal culture^25^. Staining with a fluorescent reagent, Nile Red, has been used in conjunction with FACS for detection and sorting of algal cells with high oil content^26^,^27^,^28^,^29^. For evaluation and characterization of *E. gracilis*, while FACS has been used to analyze its cell cycle by staining with propidium iodide or Hoechst 33258 after fixation^30^,^31^, it has been difficult to apply FACS to live *E. gracilis* cells due to the toxicity of the staining reagents and its low cell viability after FACS.

In this Article, we present a method that overcomes the aforementioned technical difficulties and hence enables efficient selective breeding of live oil-rich *E. gracilis* with FACS. Specifically, the breeding method is a repetitive procedure for one-week heterotrophic cultivation, staining intracellular lipids with BODIPY^505/515^, and FACS-based isolation of top 0.5% lipid-rich *E. gracilis* cells with high viability, after inducing mutation with Fe-ion irradiation to the wild type (WT). The entire breeding process only takes several weeks. As a result of using the breeding method, we acquire a live, stable, lipid-rich *E. gracilis* mutant strain, named B_1_ZFeL, with 40% more lipid content on average than the WT. We anticipate that a further understanding of lipid metabolism combined with a further optimization of the breeding method will enable rapid, cost-effective, energy-efficient production of biofuel.

## Results

### Autofluorescence of live *E. gracilis* cells

**Figure 1:**
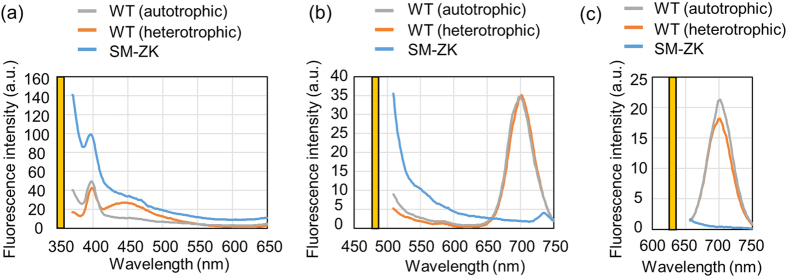
Characterization of the autofluorescence of *E. gracilis* cells. (**a–c**) Autofluorescence spectrum of the WT (Z strain) in the autotrophic and heterotrophic cultures and the chloroplast-less SM-ZK strain in the heterotrophic culture. The wavelength of the excitation laser is 350 nm (**a**), 488 nm (**b**), and 635 nm (**c**). The yellow bars indicate the wavelength of the excitation laser.

Fluorescence by staining target cells with fluorescent dyes and specific probes is commonly used for the identification of cells of interest and the key requirement for FACS. Since in the case of microalgae, their intracellular photosynthetic pigments often emit strong autofluorescence that becomes noise in FACS-based recognition of target cells, it is an essential requirement to carefully select appropriate fluorescent dyes and detection techniques^28^. To determine the requirement, we investigated the autofluorescence of *E. gracilis* cells, in particular the WT (Z strain) cultured in an autotropic and heterotrophic condition and a chloroplast-less mutant strain, SM-ZK, cultured in a heterotrophic condition when exciting them with laser light at 350 nm, 488 nm, and 635 nm (commonly used wavelengths in FACS). As shown in Fig. 1a, the excitation at 350 nm induced each strain to emit strong fluorescence at 400 nm and weak fluorescence around 450 nm. The cause of the strong fluorescence is unclear while that of the weak fluorescence is presumably NAD(P)H. Also, as shown in Fig. 1b, the excitation at 488 nm induced the WT to emit fluorescence at 700 nm. This fluorescence is caused by chlorophyll as the chloroplast-less SM-ZK strain did not emit fluorescence at this excitation wavelength. Finally, as shown in Fig. 1c, the excitation at 635 nm induced the WT to emit fluorescence at 700 nm.

### Staining of live *E. gracilis* cells with BODIPY^505/515^

**Figure 2:**
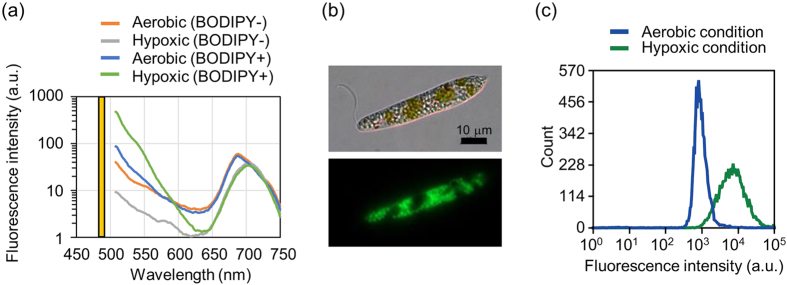
Characterization of the fluorescence of BODIPY^505/515^-stained *E. gracilis* cells. (**a**) Fluorescence spectrum of BODIPY^505/515^-stained *E. gracilis* cells. The yellow bar indicates the wavelength of the excitation laser. (**b**) DIC (upper) and fluorescent (lower) images of a BODIPY^505/515^-stained *E. gracilis* cell which was in the hypoxic condition for 2 days. (**c**) Histogram of BODIPY^505/515^-stained *E. gracilis* cells with and without hypoxic incubation obtained by flow cytometry analysis. The fluorescence intensity is the strength of the light that passed through the 529/28 bandpass filter.

Following the spectral profile of the autofluorescence from *E. gracilis* cells, we examined the capability of the lipophilic green fluorescent dye, BODIPY^505/515^, which stains neutral lipids stored in the cells just like Nile Red which is toxic and causes interference with natural cellular functions such as lipid production yield. BODIPY^505/515^ has been used to stain intracellular lipids in microalgae^32^,^33^,^34^. When excited by laser light at 488 nm, BODIPY^505/515^-stained *E. gracilis* cells emit fluorescence at 515 nm, which is compatible to the autofluorescence profile of *E. gracilis*. To test if the intracellular lipids in *E. gracilis* can be stained by BODIPY^505/515^, we incubated a heterotrophic culture of *E. gracilis* cells in a hypoxic condition to induce the conversion of paramylon to wax ester. After a sequence of condition studies, we reached an optimum staining procedure for exposing the cells to 5 μM of BODIPY^505/515^ for 5 min in water. Figure 2a shows the fluorescence spectrum of the stained cells. Here the fluorescence signal from BODIPY^505/515^-stained cells was enhanced by increasing the duration of hypoxic conditioning (Supplementary Figure 1A). The fluorescence signal was found to be well correlated with the actual lipid content (Supplementary Figures 1B,C). Also, as microscope images of the stained cells show in Fig. 2b, the cells have a granular spatial pattern of fluorescence. In contrast to staining with Nile Red, staining with BODIPY^505/515^ clearly showed the lipid granules of live cells (Supplementary Figure 2). Furthermore, to quantify the fluorescence, we performed population analysis of the stained cells with FACS. As Fig. 2c shows, the cells in the hypoxic condition show stronger fluorescence than the cells cultured in the aerobic condition. Here the fluorescence is the light that passed through the 529/28 filter.

### High-throughput sorting of live *E. gracilis* cells with high viability

Following the results about the autofluorescence and fluorescence of *E. gracilis*, we performed FACS of live *E. gracilis* cells, but with an improved nozzle. Previously, it has been difficult to conduct high-throughput sorting of *Euglena* cells with conventional cell sorters since they can easily be damaged by their nozzle with a typical diameter of 70 μm. This is presumably because *E. gracilis* does not have a cell wall, is relatively large in size, and is vulnerable to shear force compared to other microalgae. For this reason, the throughput was required to be reduced for sorting of *E. gracilis* with high viability. In our FACS analysis, more than 40% of *E. gracilis* cells were killed during the analysis and sorting process with the 70-μm nozzle (Supplementary Figure 3A). In order to minimize the damage to the cells, we investigated and determined the optimum conditions for sorting live *E. gracilis* cells using multi-well plates filled with KH medium with nozzles of 70 μm, 100 μm, and 120 μm in diameter. After two weeks of static cultivation for proliferation, we enumerated the wells in which the cells survived and determined the survival rate of the cells. Only 57% of the wells (110 wells/192 wells) showed proliferation with the 70-μm nozzle while 97% (187 wells/192 wells) and 99% (190 wells/192 wells) were recovered with the 100-μm and 120-μm nozzles, respectively (Supplementary Figure 3B).

### Production of a lipid-rich *E. gracilis* mutant strain

**Figure 3:**
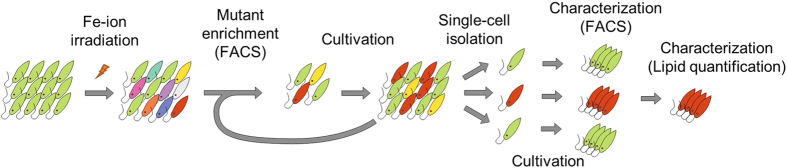
Method for selective breeding of a lipid-rich *E. gracilis* mutant strain. The procedure of the method is as follows. (i) A pool of WT (Z strain) *E. gracilis* cells are irradiated with a Fe-ion beam for mutation induction. (ii) Mutants are enriched by isolation with FACS and BODIPY^505/515^ staining. (iii) The selected mutants are cultivated for a week. (iv) The process of the mutant enrichment and cultivation is repeated four times. (v) After four rounds of the mutant enrichment and cultivation, 15 individual mutants are randomly isolated and cultivated independently. (vi) Each established mutant strain is analyzed by FACS, such that the best stained strain is selected for further characterization. (vii) The lipid content of the selected strain is quantified. The entire breeding process only takes several weeks.

The results from lipid staining with BODIPY^505/515^ and high-throughput sorting suggest that FACS is an effective high-throughput tool for selectively breeding *E. gracilis* based on its lipid content. To demonstrate this, we designed a screening procedure for enriching mutants which produce wax ester even in the aerobic culture condition (Fig. 3). Specifically, *E. gracilis* cells were irradiated by a 50 Gy Fe-ion beam to induce mutation and were then separated to four independent groups, each of which consists of 10^5 ^cells. Each group was let recover for a week in a heterotrophic condition to accumulate paramylon (which was converted to wax ester in the mutants) and then stained with BODIPY^505/515^ and subjected to cell sorting. The top 0.5% of the total population of the stained cells with the BODIPY^505/515^ fluorescence signal was collected and cultured in the same condition. After repeating this procedure for enriching the mutants 4 times with a one-week interval between the consecutive sorting steps, 15 mutant candidates were randomly isolated from each mutant pool. The phenotype of the progenies of each candidate was evaluated by FACS with BODIPY^505/515^ staining. Finally, we selected the strain with the largest fluorescence strength, which we named B_1_ZFeL after the nomenclature^16^ with the addition of the phenotypic designation “B” for BODIPY^505/515^ staining and the mutagen designation “Fe” for Fe-ion irradiation, and applied it to further phenotypic characterization.

**Figure 4:**
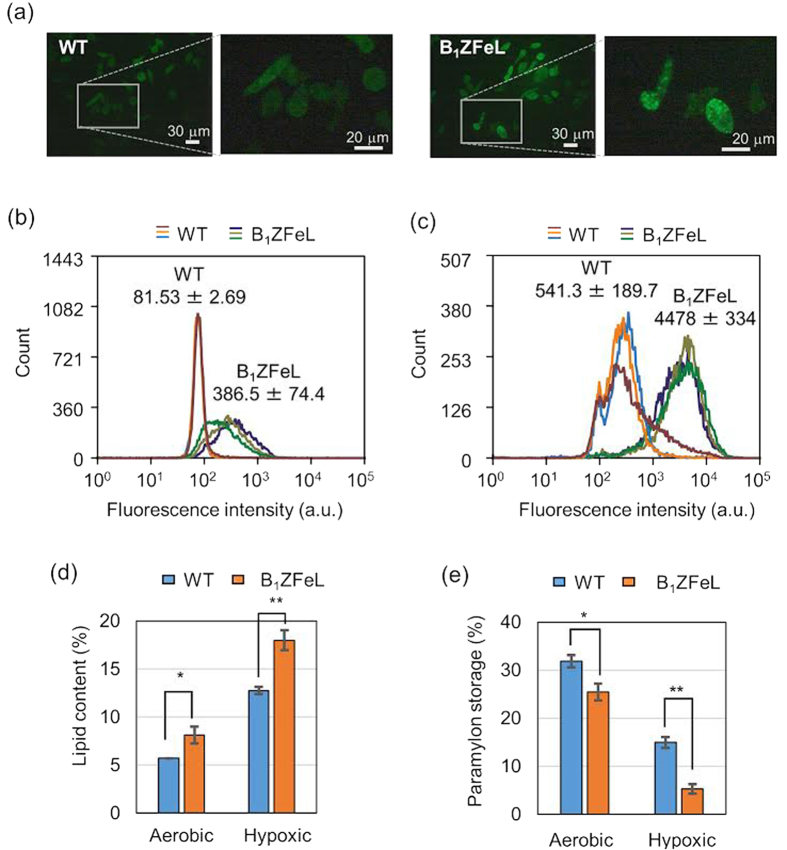
Characterization of the lipid-rich strain B_1_ZFeL. (**a**) Fluorescence images of BODIPY^505/515^-stained WT (Z strain) and B_1_ZFeL cells in the aerobically heterotrophic culture. (**b,c**) Histogram of the BODIPY^505/515^-stained WT and B_1_ZFeL cells. The cells without (**b**) and with (**c**) hypoxic incubation were stained with BODIPY^505/515^ and analyzed by FACS analysis. The fluorescence intensity is the strength of the light that passed through the 529/28 bandpass filter. The three histograms represent the results of independent cultures for each strain. The values in the plots are the average fluorescence value of each strain with the standard error of the mean N = 3. (**d,e**) Lipid (**d**) and paramylon (**e**) contents of the WT and B_1_ZFeL in the heterotrophic culture with and without hypoxic incubation. N = 3. *p < 0.05, **p < 0.005, *t*-test with Bonferroni’s correction.

As the fluorescence microscope images in Fig. 4a show, the B_1_ZFeL strain showed granule-like stained properties without hypoxic conditioning while the growth rate of the B_1_ZFeL strain in a conical flask was found to be slightly lower than that of the WT (Z strain) (Supplementary Figure 4). As shown in Fig. 4b, the B_1_ZFeL strain shows a factor of 4.7 stronger fluorescence in our flow cytometry analysis (FACS measurements without cell sorting) after BODIPY^505/515^ staining than the WT. Here the values in the figure indicate the mean and the standard error of the mean of each strain. As shown in Fig. 4c, after hypoxic incubation, the fluorescence intensity of both the WT and B_1_ZFeL distributions increased while the difference between the two strains increased to a factor of 8.3.

In order to directly quantify the actual neutral lipid content of the harvested cells, we measured the weight proportion of the neutral lipids extracted by n-hexane. As shown in Fig. 4d, the results are consistent with those from the flow cytometry analysis with BODIPY^505/515^-stained *E. gracilis* cells. It is shown that the B_1_ZFeL mutant has 1.4 times higher lipid content than the WT both in the aerobic condition and after the hypoxic incubation. Finally, to study the underlying mechanism of the high lipid content, we also examined the weight proportion of paramylon in the cells. As shown in Fig. 4e, the intracellular content of paramylon was found to be lower in the B_1_ZFeL mutant than the WT both in the aerobic condition and after the hypoxic incubation. The increase in the B_1_ZFeL mutant’s lipid content accompanied by the decrease in its paramylon storage suggests that the higher lipid content is derived from a constantly enhanced wax fermentation pathway in the cell. Also, the proportion of fatty acids and alcohols which account for the extracted lipids were found to be nearly identical in the WT and B_1_ZFeL mutant (Supplementary Figures 5A,B, Supplementary Tables 1A,B). These results indicate that the B_1_ZFeL mutant’s phenotype was not derived from a malfunction of the specific lipid metabolic pathway, but from a general upregulation of the wax fermentation pathway.

## Discussion

In our proof-of-principle demonstration of the breeding method, we obtained the industrially useful mutant strain, B_1_ZFeL, with 40% more lipid content than the WT. The produced strain was found to store wax ester at the expense of lower paramylon storage, which implies the constant upregulation of the wax fermentation process in the mutant strain. The BODIPY^505/515^-based fluorescence was 4.7 and 8.3 times higher in the B_1_ZFeL strain than the WT in the aerobic condition and after the hypoxic incubation, respectively, whereas the neutral lipid content was measured to be only 1.4 times higher in the B_1_ZFeL than the WT both in the aerobic condition and after the hypoxic incubation. These discrepancies are presumably due to the reason that either the BODIPY^505/515^-stained granules include only a fraction of the entire lipid content that was extracted by n-hexane or the B_1_ZFeL mutant has an additional phenotype that results in a higher permeability for the staining reagents.

It is worthwhile to stress that while B_1_ZFeL mutants showed the higher lipid content than the WT in the aerobic condition, the lipid content further increased after the hypoxic incubation. Such a phenotypic alteration may be accomplished by modifying its environment recognition mechanism. B_1_ZFeL may have an incompletely altered recognition of the environment, meaning that the cells recognize the aerobic condition as a hypoxic condition, which possibly manifests a weak allele of the predetermined target gene. While the obtained mutant also requires a wax fermentation process for an efficient conversion of paramylon into lipid, the efficiency of the process can be enhanced significantly by using the strain. In our demonstration, we performed screening of mutants from four groups of 10^5^ independent genomes (cells) modified by the Fe-ion irradiation, but in order to obtain better mutant strains, it is desirable to expand the size of screening with the same process.

The wax ester fermentation process is an energy-generating reaction in which ATP is produced in the process of decomposing glucose, which is formed by the hydrolysis of paramylon, into pyruvic acid. The following *E. gracilis-*specific fatty acid synthesis pathway in mitochondria to form acyl CoA from pyruvic acid is break-even in total^13^,^35^. Because the paramylon storage was decreased in B_1_ZFeL (Fig. 4e), its primary carbon source is considered to be the paramylon or surplus glucose. However, some of the glucose which was to be supplied to the TCA cycle may also be used for the wax production because the B_1_ZFeL mutant showed a slower growth than the WT (Supplementary Figure 4). In fact, on the 6th day of heterotrophic cultivation, the number of B_1_ZFeL cells was about 85% of that of the WT while the lipid content after the hypoxic incubation was 1.4 times higher in B_1_ZFeL mutants (Fig. 4d). These numbers indicate that even after the same duration of cultivation, the B_1_ZFeL mutant strain yields an about 20% higher lipid amount with an increased productivity.

Finally, the utility of our FACS method is not limited to the live cell sorting of *E. gracilis*, but is also applicable to other *Euglena* species. Other than *E. gracilis*, there exist more than 800 *Euglena* species and related species with common traits of accumulating paramylon and wax^36^. While *E. gracilis* is unique in that they proliferate at a high rate, many of the other species also have characteristics useful for industrial applications. For example, *E. anabaena* var. *minor* precipitates easily^37^, *E. mutabilis* grows in acid mine drainages^38^, and *E. sanguinea* produces carotenoid astaxantin^39^. While not included in this Article, we have attempted and verified that FACS analysis can also be applied to *E. anabaena* var. *minor* and *E. mutabilis*. While *E. gracilis* is currently the only *Euglena* species available for industrial use and other species still have a number of technical and economical difficulties to overcome before industrial use, selective breeding of such species is expected to broaden the utility of *Euglena*.

## Materials and Methods

### Strains and culture media

The *E. gracilis* strains used in the study are a WT Z strain and chloroplast-less SM-ZK strain^40^ originally provided by IAM (JAPAN) and maintained at euglena Co., Ltd. for 10 years. The *E. gracilis* Z strain were maintained in CM medium (pH 3.5)^41^ while the *E. gracilis* SM-ZK strain was maintained in KH medium (pH 3.5)^42^. For an autotrophic culture and heterotrophic culture of *E. gracilis*, CM medium and KH medium were used, respectively.

### Autofluorescence measurements

The autofluorescence measurements were performed on the *E. gracilis* WT Z strain cultured in CM medium or KH medium and the chloroplast-less SM-ZK strain cultured in KH medium. Each culture was centrifuged (2,000 g, 30 sec) and washed by water three times. A sample of 10^5^ *E. gracilis* stain cells was diluted to 1 mL in water. The fluorescence from the strains was measured by a fluorophotometer (F-2500, Hitachi) with excitation light at 350 nm, 488 nm, or 635 nm. For the autofluorescence measurements of BODIPY^505/515^-stained cells, the cells with and without hypoxic incubation were used. The hypoxic incubation was performed by filling a 1.5 mL tube with the cells at a concentration of 10^7 ^cells/mL in the KH medium and incubating the tube in the dark for 2 days at 23 °C.

### BODIPY^505/515^ staining for fluorescence measurements

The stock solution of BODIPY^505/515^ was provided by dissolving it to DMSO at 1 mM. The stock solution was diluted in water to 10 μM just before use. The cells for staining were cultured as noted. The centrifuged cells at 2,000 g for 30 sec were suspended in water. 200 μL of the prepared 10 μM BODIPY^505/515^ solution was added to the 200 μL cell suspension which includes 2 × 10^5^ cells in water. The mixture was gently mixed and incubated in the dark for 5 min. Then, the cells were washed with water three times by centrifuging the sample at 2,000 g for 30 sec and re-suspended in water. The stained cells were protected from light until the time of use.

### FACS

A fluorescence-activated cell sorter, MoFlo XDP (Beckman Coulter), was used for FACS analysis and cell sorting. This FACS machine is equipped with two semiconductor lasers at wavelengths of 488 nm (Sapphire, Coherent) and 642 nm (CUBE, Coherent) which was not used. The fluorescence signal from each cell was dissected to each wavelength range by optical filters and detected by a series of photomultiplier tubes (H957-27, HAMAMATSU). The fluorescence from BODIPY^505/515^-stained cells was evaluated by the FACS machine’s FL1-log-height value, which represents the fluorescence intensity that passed through a 529/28 bandpass filter. As described above, the 100-μm nozzle tip was used for high-throughput sorting of *E. gracilis*. To obtain unbiased results from FACS tests, the sample needed to be tested as soon as it was mixed and set to the suction tube of the FACS machine because wax-ester-rich cells are smaller in specific gravity than paramylon-rich cells which tend to precipitate. Otherwise, the proportion of wax-ester-rich cells to the overall sample volume would vary over the measurement duration. For this reason, every sample was well mixed before it was set to the FACS machine and subjected to FACS tests. The acquisition of FACS data was completed within a minute. Cell sorting was performed with suspending the precipitated cells every few minutes.

### Fe-ion irradiation

Fe-ion irradiation was conducted in the same way as in a previous report^19^ with minor modifications. *E. gracilis* cells were mutagenized by Fe-ion irradiation. 1 mL of *E. gracilis* cells (4 × 10^5^ cells/mL) in CM medium were encapsulated in 5 × 7 cm sized hybridization bags and irradiated by Fe ions (LET: 650 keV/μm) at 50 Gy in the RIKEN RI-beam factory (Wako, Saitama, Japan; http://www.rarf.riken.go.jp/Eng/facilities/RIBF.html). The cultures were let recover for a week in 1 mL of KH medium on 24 well plates before being subjected to the mutant screen. The recovery cultures were put under 100 μmol photons/m^2^/sec of constant illumination at 29 °C.

### Growth test

The growth test on *E. gracilis* cells in the heterotrophic condition was conducted by culturing them in conical flasks on a rotary shaker with about 100 μmol photons/m^2^/sec of constant illumination at 29 °C. The strength of light illuminated on each flask was averaged by changing the positions of the conical flasks every day. The cells precultured in KH medium were washed. The culture was started with 10^6 ^cells in 100 ml of KH medium (OD680 ≈ 0.09). The cell density was measured by a particle analyzer (CDA-1000, Sysmex) each day. On the 6^th^ day, half of the culture was harvested by centrifugation (2,000 g, 2 min) for FACS analysis and quantification of the intracellular paramylon and lipid content. The other half was subjected to hypoxic incubation by sealing the top of the conical flask and protected from ambient light. After 4 days of hypoxic incubation, the remaining half was harvested and analyzed in the same way.

### Quantification of paramylon and lipids

The intracellular paramylon and lipids were quantified to verify the changes in their content. The harvested cells were dried in a freeze dryer (FDV-1200, EYELA). Approximately 10 mg and 100 mg of samples of dried cells were used for quantifying their intracellular paramylon and lipid content, respectively. The paramylon content was evaluated in the same way as in previous reports^13^,^37^. The dried cells were briefly suspended in 10 mL of acetone and homogenized twice for 90 sec by using a sonicator (UD-201, TOMY). The extract was collected by centrifugation (800 g, 5 min), boiled for 30 min in 10 mL of 1% sodium dodecyl sulfate aqueous solution, and washed twice with 10 mL of 0.1% sodium dodecyl sulfate aqueous solution and then with water. The extracted insoluble carbohydrate, which consists almost entirely of paramylon in *E. gracilis*^13^, was quantified using the phenol-sulfuric acid method^43^. Likewise, The neutral lipid was extracted from the dried cells by using traditionally used n-hexane as a solvent^44^. 10 mL of n-hexane was added to the dried cells. The suspending solution was homogenized for 90 sec by using a sonicator (UD-201, TOMY). The liquid phase was collected by filtering it with a piece of glass fiber filter paper. Another round of hexane extraction was conducted on the residue. The collected lipid dissolving hexane was evaporated using a rotary evaporating system (N-1100 and NVC-2100, EYELA). After drying, the residue’s weight left in the flask was quantified as the extracted total neutral lipid.

## Additional Information

**How to cite this article**: Yamada, K. *et al*. Efficient selective breeding of live oil-rich *Euglena gracilis* with fluorescence-activated cell sorting. *Sci. Rep.* **6**, 26327; doi: 10.1038/srep26327 (2016).

## Supplementary Material

Supplementary Information
