# Supplementary material for: Efficient selective breeding of live oil-rich Euglena gracilis with fluorescence-activated cell sorting
Source: Sci Rep. 2016 May 23;6:26327. doi: 10.1038/srep26327 (PMC4876468; doi:10.1038/srep26327)
Supplement: Supplementary Information [file srep26327-s1.pdf]

# Supplementary Information

## Efficient selective breeding of live oil-rich *Euglena gracilis* with fluorescence-activated cell sorting

Koji Yamada[1], Hideyuki Suzuki[1], Takuto Takeuchi[1], Yusuke Kazama[2], Sharbanee Mitra[1], Tomoko Abe[2], Keisuke Goda[3,4,5*], Kengo Suzuki[1], & Osamu Iwata[1,*]

(A) (B) (C)

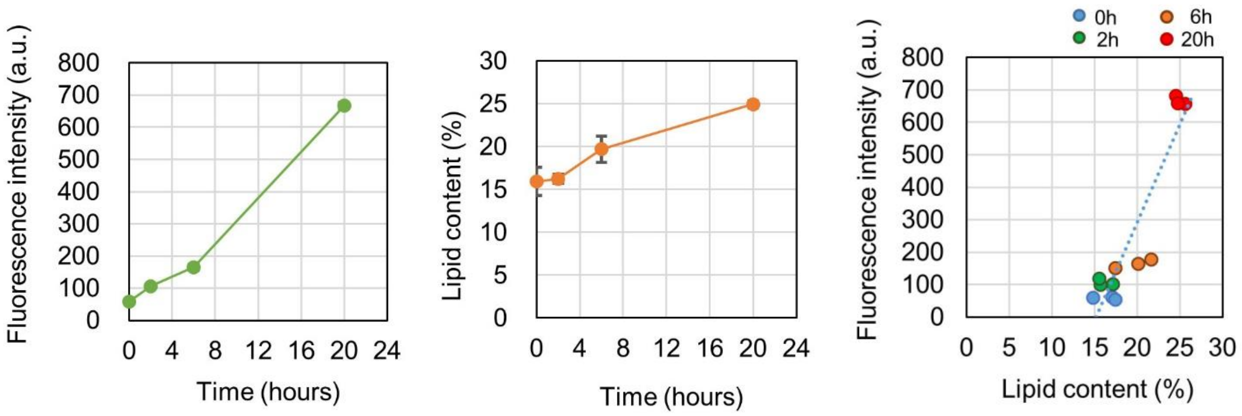


**Supplementary Figure 1. Correlation between the BODIPY$^{505/515}$ fluorescence and intracellular lipid content.** (A-C) *E. gracilis* WT cells cultured in KH medium were harvested and subjected to 2, 6, and 20 hours of hypoxic incubation by packing them in 50 mL conical tubes at $4 \times 10^6$ cells/mL and incubating them at 29°C with light shielding. BODIPY$^{505/515}$ staining was performed as noted in the main text. The fluorescence from $10^5$ cells at 515 nm was quantified by a fluorophotometer (F-2500, Hitachi) with excitation light at 488 nm. N = 3 (A). In addition, the intracellular lipid was extracted by hexane and quantified. N = 3 (B). The BODIPY$^{505/515}$ fluorescence and lipid content were plotted for each sample (C).

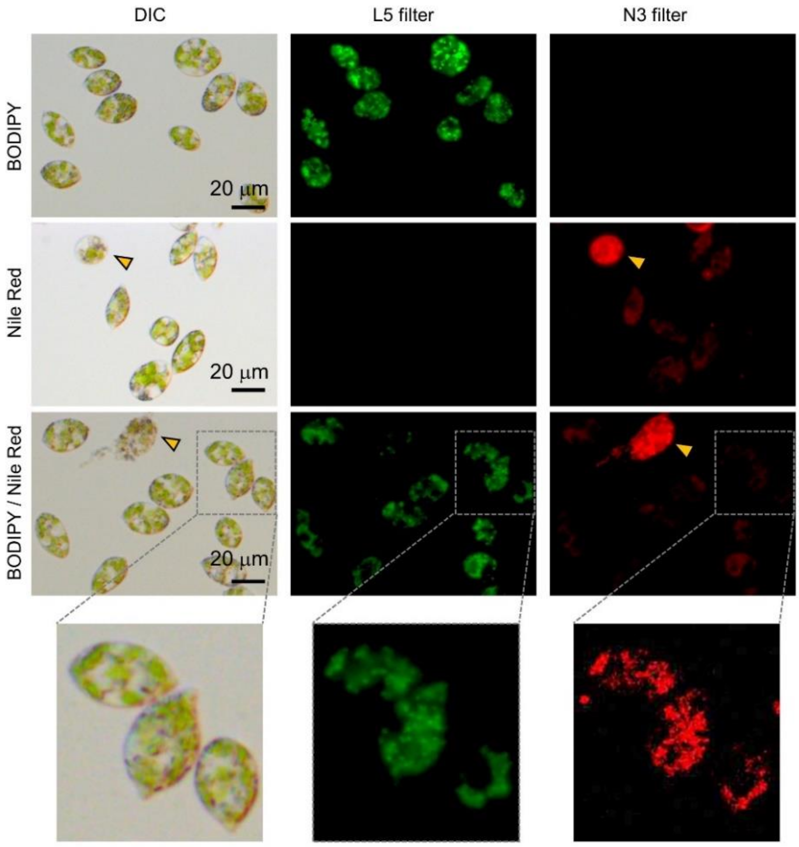


**Supplementary Figure 2. Lipid staining with BODIPY$^{505/515}$ and Nile Red.** *E. gracilis* cells under the hypoxic condition were stained with 5 µM BODIPY$^{505/515}$ and/or 5 µg/mL Nile Red. The images show that Nile Red staining worked strong on dead cells (indicated by the yellow arrowheads) while BODIPY$^{505/515}$ stained live cells. In the enlarged images, staining of the same cellular compartments with BODIPY$^{505/515}$ and Nile Red can be seen. The brightness of the enlarged images was adjusted to compare the stained intracellular region between the two staining methods. Fluorescent dyes were excited by the light that passed through 480/40 and 546/12 band-pass filters while the fluorescence that passed through 527/30 and 600/40 band-pass filters was observed with L5 and N3 filter cubes (Leica), respectively.

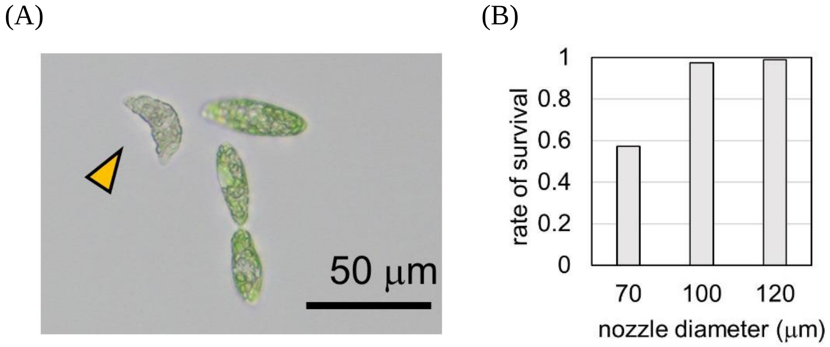


**Supplementary Figure 3. Effect of different nozzle sizes on the survival rate of *E. gracilis*.** (A) Microscope image of cells after high-throughput sorting with the 70-µm nozzle. The arrowhead indicates a dead cell after sorting. (B) Survival rate of cells after high-throughput sorting for three different nozzle sizes. After isolating 192 *E. gracilis* cells in 96-well plates filled with KH medium, the wells with cells that show proliferation were enumerated. The rate of survival was calculated by dividing the number of wells in which the cells proliferated by 192.

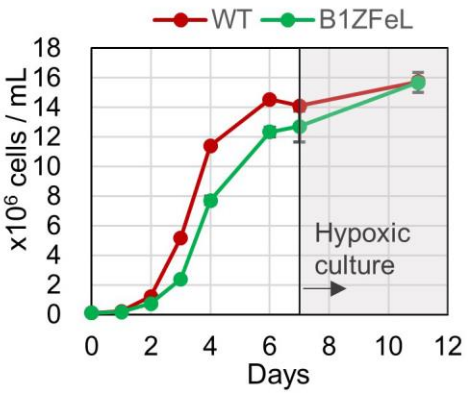


**Supplementary Figure 4. Growth curves of the WT and B1ZFeL in the heterotrophic culture in separate conical flasks.** The WT and $B_1$ZFeL were cultured in conical flasks in KH medium. On the $7^{th}$ day, half of the culture was harvested while the rest was subjected to hypoxic incubation by sealing the top of the conical flasks and protecting it from ambient light. N = 3. The error bars indicate the standard error of the mean.

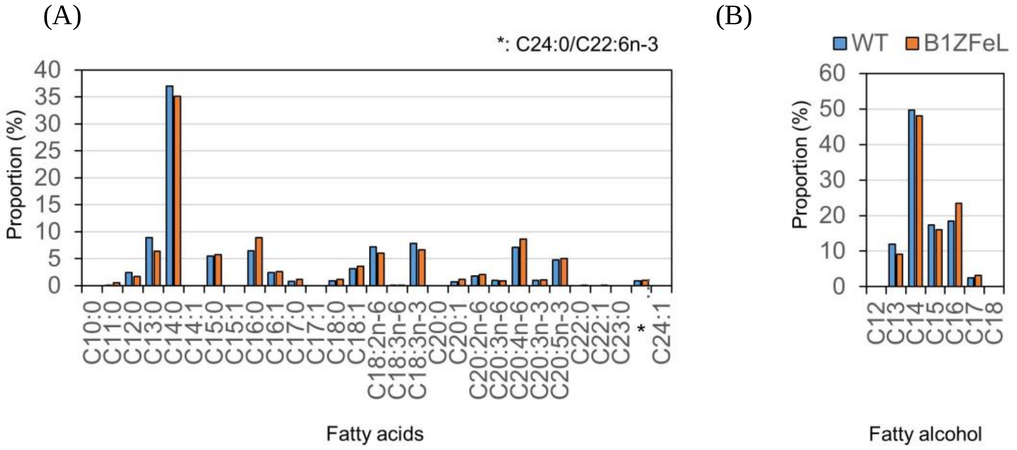


**Supplementary Figure 5. Proportion of fatty acids and alcohol in the extracted neutral lipids.** (A and B) Neutral lipids were processed by 5% HCl/MeOH at 70°C for 3 hours to convert to FAME, fatty acid methyl ester, and fatty alcohol. The decomposed matter was collected by n-hexane, evaporated, and then re-dissolved in chloroform. The resulted solution was subjected to GC-MS analysis and quantified each ingredients by using methyl stearate as an internal standard. The proportion of fatty acids (A) and fatty alcohols (B) in *E. gracilis* WT Z strain and $B_1$ZFeL which were incubated in hypoxic condition were displayed. The rates were calculated by dividing the estimated weight of each components by that of total.

(A)

| fatty acid | Proportion (%) WT | B1ZFeL |
|---|---|---|
| C10:0 | - | - |
| C11:0 | 0.1 | 0.5 |
| C12:0 | 2.4 | 1.7 |
| C13:0 | 8.9 | 6.4 |
| C14:0 | 37 | 35.1 |
| C14:1 | - | - |
| C15:0 | 5.5 | 5.8 |
| C15:1 | - | - |
| C16:0 | 6.5 | 8.9 |
| C16:1 | 2.4 | 2.6 |
| C17:0 | 0.8 | 1.2 |
| C17:1 | - | - |
| C18:0 | 0.9 | 1.2 |
| C18:1 | 3.1 | 3.6 |
| C18:2n-6 | 7.2 | 6 |
| C18:3n-6 | 0.1 | 0.1 |
| C18:3n-3 | 7.8 | 6.7 |
| C20:0 | - | 0 |
| C20:1 | 0.7 | 1.2 |
| C20:2n-6 | 1.8 | 2.1 |
| C20:3n-6 | 1 | 0.9 |
| C20:4n-6 | 7.1 | 8.6 |
| C20:3n-3 | 1 | 1.1 |
| C20:5n-3 | 4.8 | 5 |
| C22:0 | - | 0.1 |
| C22:1 | - | 0.1 |
| C23:0 | - | - |
| * | 0.9 | 1 |
| C24:1 | - | - |
| total | 100 | 100 |

(B)

| fatty alcohol | Proportion (%) WT | B1ZFeL |
|---|---|---|
| C12 | - | - |
| C13 | 12.0 | 9.2 |
| C14 | 49.7 | 48.1 |
| C15 | 17.4 | 16.0 |
| C16 | 18.4 | 23.5 |
| C17 | 2.5 | 3.2 |
| C18 | - | - |
| total | 100 | 100 |

*: C24:0/C22:6n-3

-: not detected

**Supplementary Table 1. Proportion of intracellular fatty acids and fatty alcohols.** (A and B) The numeric values shown in the tables (A) and (B) are the specific values of data shown in Supplementary Figure 5A and Supplementary Figure 5B, respectively.
